# Pacemaker implantation through pericardial reflections under fluoroscopic guidance: a novel approach for patients with limited venous access

**DOI:** 10.1186/1749-8090-8-S1-O58

**Published:** 2013-09-11

**Authors:** R Costa, M Scanavacca, KR Silva, M Martinelli Filho, MS Lacerda, RM Oliveira, ES Crevelari, FB Jatene

**Affiliations:** 1 Cardiovascular Surgery Department, Heart Institute (InCor), Hospital das Clínicas da Faculdade de Medicina da Universidade de São Paulo, São Paulo, Brazil

## Background

The purpose of this study was to describe a novel approach for epimyocardial pacemaker implantation under fluoroscopic guidance associated to atrial access through pericardial reflections as an alternative technique for lead implantation in patients with limited venous access.

## Methods

Between June 2006 and November 2011, 15 adult patients underwent epimyocardial atrioventricular pacemaker implantation through a minimally invasive subxiphoid approach and pericardial window. Mean age was 46.4 ± 15.3 years and 9 (60.0%) patients were male. Patients selected for this new surgical approach were not amenable to transvenous lead placement due to: multiple abandoned leads (5), venous occlusion (3), presence of retained lead fragment in the intravascular after previous device extraction (3), tricuspid valve vegetation under treatment (2) and uncorrected intracardiac defects (2).

## Results

All procedures were successfully performed. There were no perioperative complications and no early deaths. The mean operating time for isolated pacemaker implantation was 231.7± 33.5 minutes. Lead placement on the roof of right atrium through the transverse sinus was possible in 12 patients and in 3 patients the atrial lead was implanted on the left atrium through the oblique sinus. None of the patients displayed pacing or sensing dysfunctions and all parameters remained stable throughout the follow-up period of 36.8 ± 25.1 months.

## Conclusion

Epimyocardial pacemaker implantation under fluoroscopic guidance associated to atrial access through pericardial reflections provides a safe, effective and reproducible approach for atrioventricular pacing in patients for whom the transvenous approach is undesirable or not feasible.

